# Supplementary figures and images for: Prognostic value of neoadjuvant therapy for resectable and borderline resectable pancreatic cancer: A meta-analysis of randomized controlled trials
Source: PLoS One. 2023 Sep 6;18(9):e0290888. doi: 10.1371/journal.pone.0290888 (PMC10482298; doi:10.1371/journal.pone.0290888)

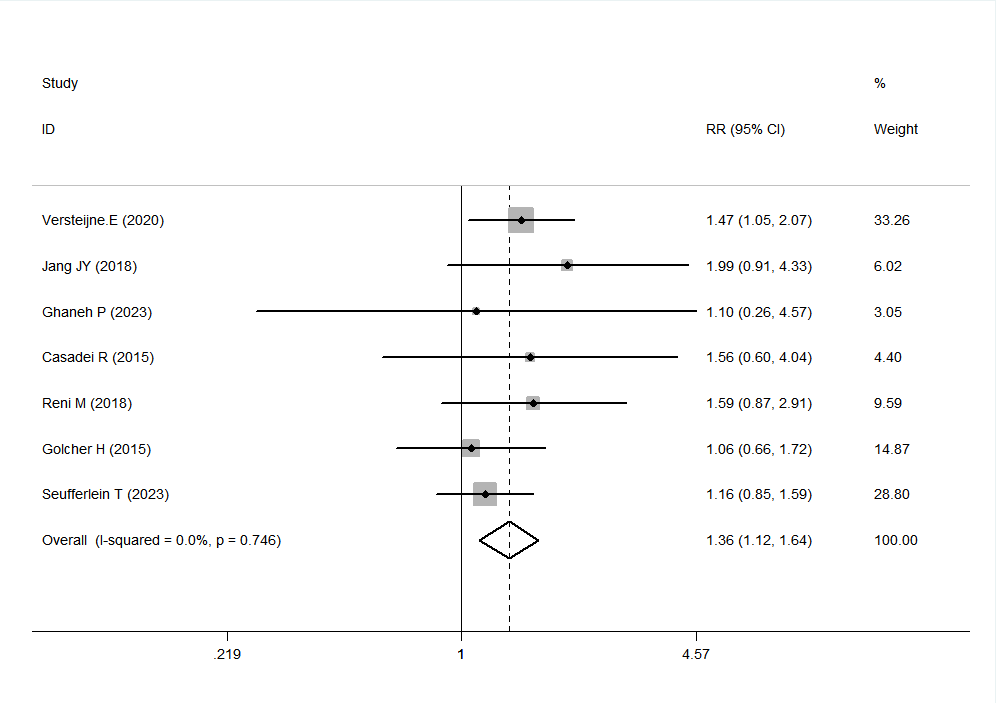

Supplement: S1 Fig — (TIF) [file pone.0290888.s002.tif]

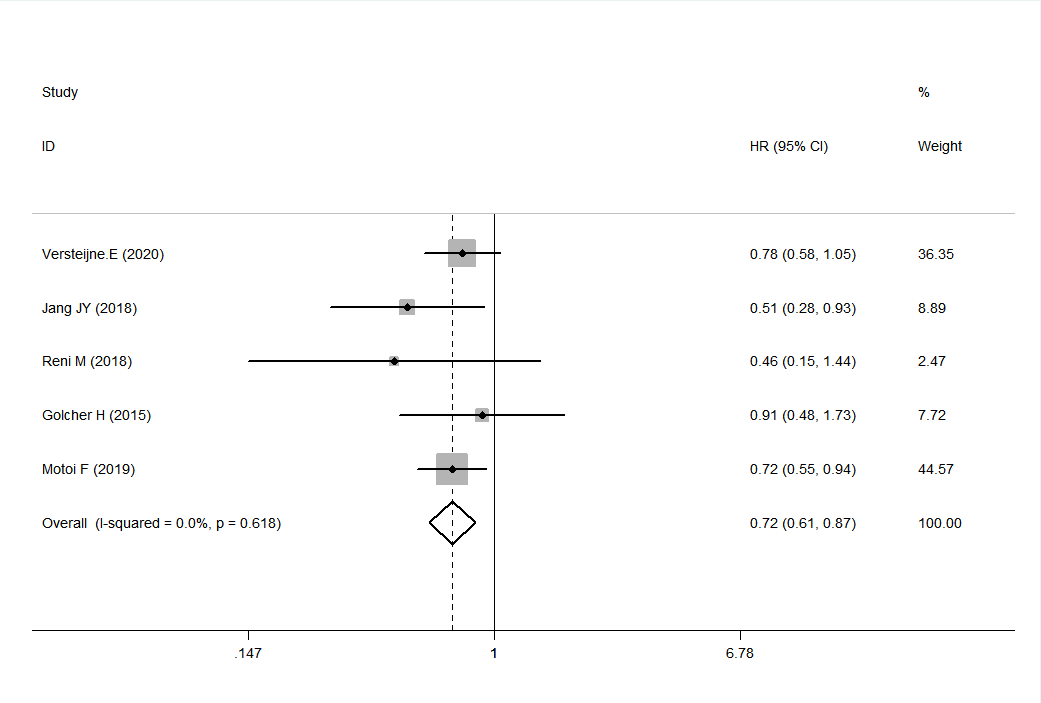

Supplement: S2 Fig — (TIF) [file pone.0290888.s003.tif]

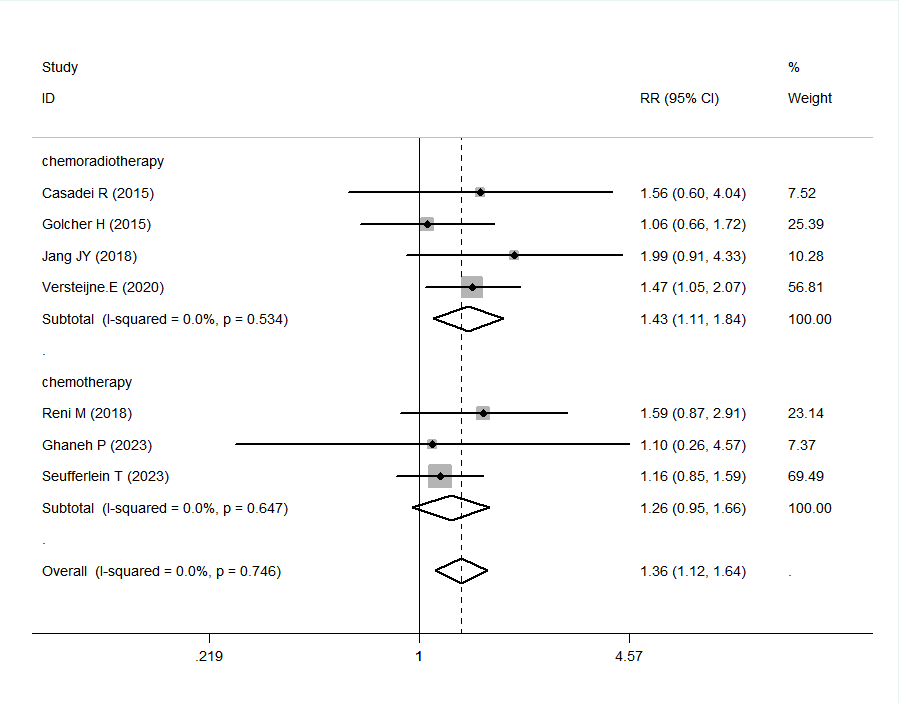

Supplement: S3 Fig — (TIF) [file pone.0290888.s004.tif]

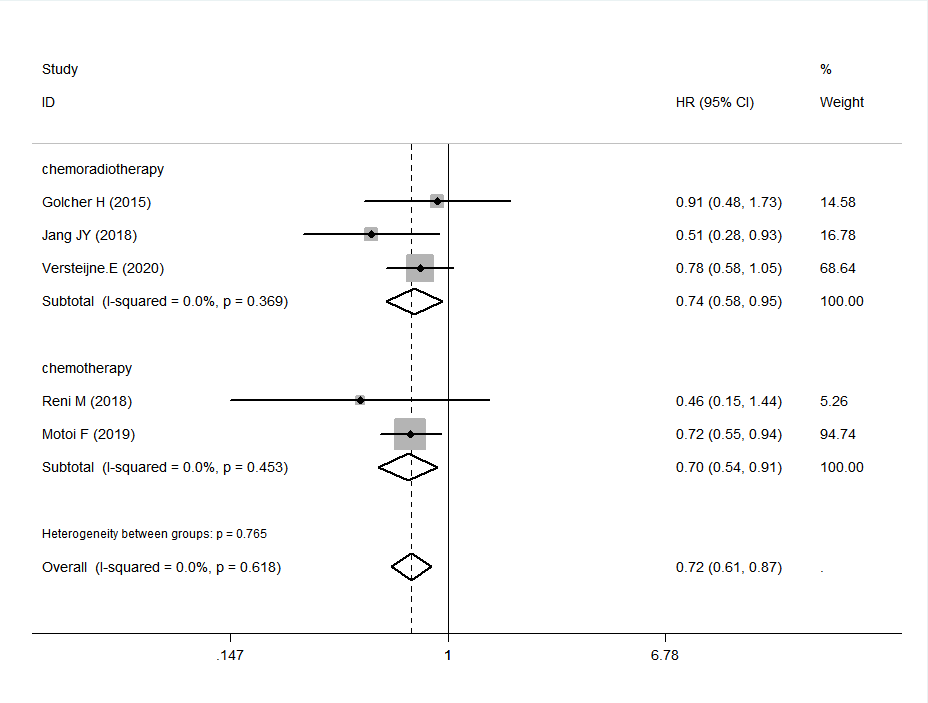

Supplement: S4 Fig — (TIF) [file pone.0290888.s005.tif]

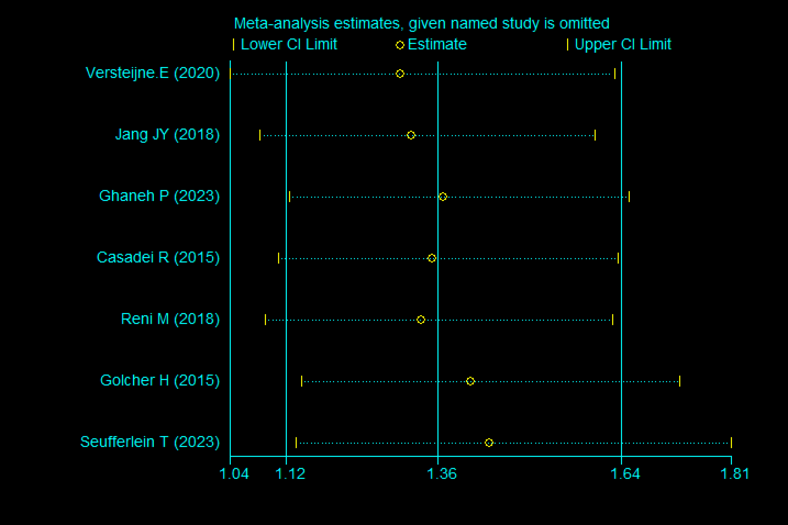

Supplement: S5 Fig — (TIF) [file pone.0290888.s006.tif]

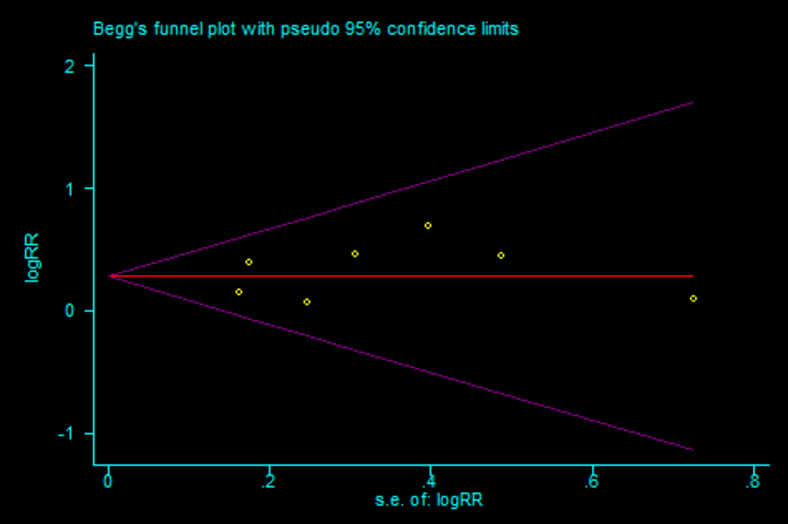

Supplement: S6 Fig — (TIF) [file pone.0290888.s007.tif]

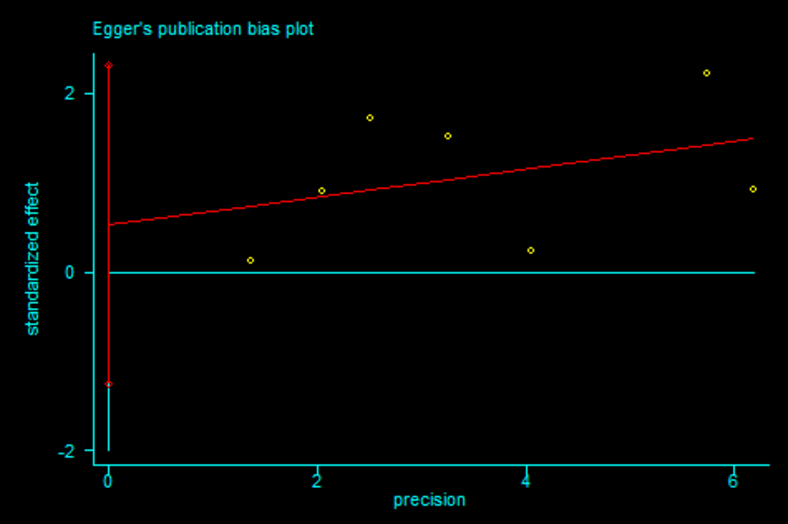

Supplement: S7 Fig — (TIF) [file pone.0290888.s008.tif]

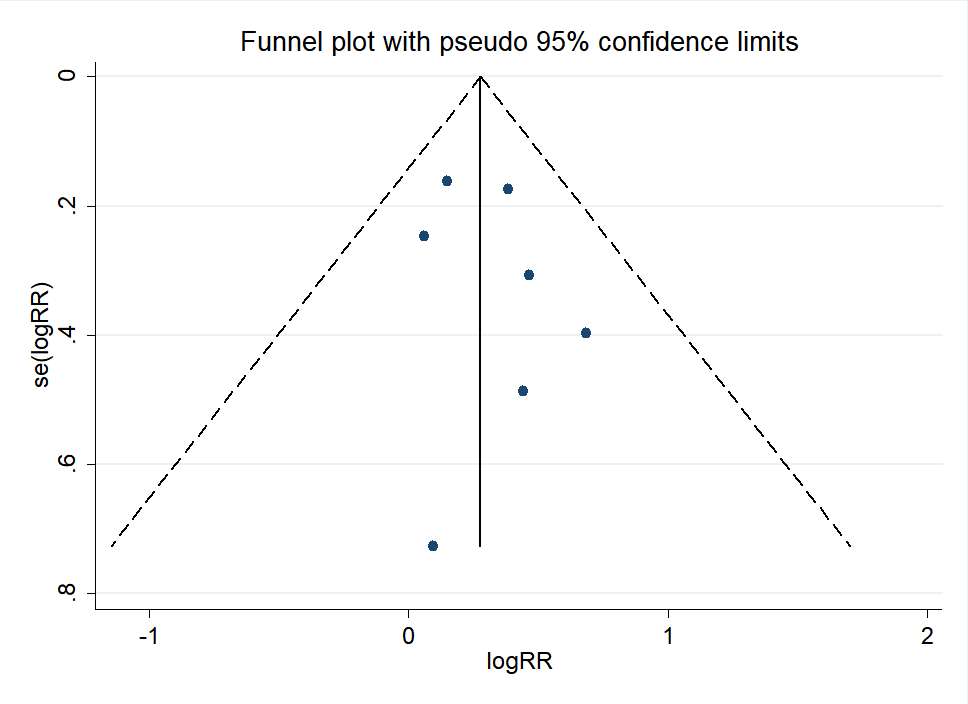

Supplement: S8 Fig — (TIF) [file pone.0290888.s009.tif]
