# Supplementary material for: Prognostic value of neoadjuvant therapy for resectable and borderline resectable pancreatic cancer: A meta-analysis of randomized controlled trials
Source: PLoS One. 2023 Sep 6;18(9):e0290888. doi: 10.1371/journal.pone.0290888 (PMC10482298; doi:10.1371/journal.pone.0290888)
Supplement: Attachment — Submitted filename: rev.docx [file pone.0290888.s010.docx]

The authors of this meta-analysis discuss the role of neoadjuvant RT in the context of resectable and borderline resectable pancreatic cancer.

Introduction.

- When the authors comment on the variability of prognosis in the context of resectable patients receiving the same treatment, I would add the reference.
- In this context, the authors talk about the need to look for biological markers of resectability, but I would ask the authors to discuss the need to look for markers of aggressiveness and thus different prognosis.
- I would remove sentences and references regarding the role of neoadjuvant treatments in oesophagus and breast from the introduction.

Data and methods

- No specific comment

Outcomes

- Is there a benefit over R0 in resectable patients with RT or chemoradiotherapy?

Discussion

- The authors only mention R0 and OS. Why these outcomes and not local control and distant metastasis free survival? These are also indicators that may differ depending on neoadjuvant therapy and have an impact on OS and quality of life.
- I would like to comment on the mechanism of alteration of the pancreatic tumour microenvironment described in Farren's study.
- Did the authors evaluate the effect of adjuvant therapies?
- What is the dose and fractionation of radiotherapy?
- Add an explanation of the acronyms 'R' and 'BR' in the legend of Table 2.
